# Peri-Contrast Staining as a Marker of Stent Failure: Restenosis, Thrombosis, and Fracture

**DOI:** 10.1155/2021/4688228

**Published:** 2021-09-06

**Authors:** Fakilahyel S. Mshelbwala, Brittany Fuller, Khaldoon Alaswad, Mir B. Basir

**Affiliations:** ^1^Cardiology Fellow PGY4, Henry Ford Hospital, K-14 Cardiology, 2799 W. Grand Blvd, Detroit, MI 48202, USA; ^2^Department of Cardiology, Henry Ford Hospital, Detroit, Michigan, USA

## Abstract

Peri-stent contrast staining (PSS), defined as contrast staining around the stent struts, has been identified as an indicator of future stent failure in first generation, sirolimus-eluting coronary stents. 1 PSS has been associated with in-stent restenosis, stent thrombosis, stent fracture, and the development of coronary aneurysm. As the frequency of patients with first generation sirolimus-eluting coronary stents becomes infrequent; PSS may go unrecognized. Herein, we present a patient with a decade of longitudinal follow-up, who developed PSS identified on coronary angiogram with recurrent stent failure.

## 1. Introduction

First generation, sirolimus-eluting coronary stents, such as the Cypher stent (Cordis Corp., Miami Lakes, Florida), were approved for use by the FDA in 2003. Peri-stent contrast staining (PSS), defined as contrast staining around the stent struts, has been identified as an indicator of future stent failure in such stents. 1 PSS has been associated with in-stent restenosis, stent thrombosis, stent fracture, and the development of coronary aneurysm. As the frequency of patients with first generation sirolimus-eluting coronary stents becomes infrequent, PSS may go unrecognized. Herein, we present a patient with a decade of longitudinal follow up, who developed PSS which was identified on coronary angiogram with recurrent stent failure.

## 2. Case Report

**Figure 1:**
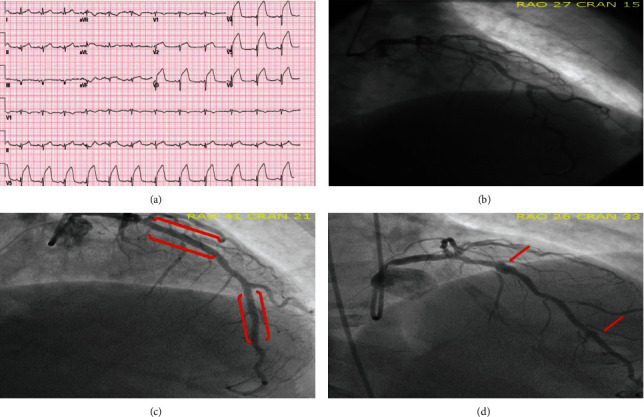
(a) ECG on presentation for the current admission showing ST segment elevation in V3-V6. (b) Initial angiogram on presentation in 2008. The patient had presented with an inferior wall myocardial infarction and underwent primary PCI of the RCA and subsequent staged PCI of the LAD one month later. (c) Angiogram after staged PCI of the LAD using 3.5 mm × 28 mm Cypher stent in the proximal segment of the LAD and overlapping 3 mm × 28 mm and 2.75 mm × 18 mm Cypher stents in the mid to distal LAD (stent locations marked with red brackets). (d) NSTEMI in 2011 secondary to thrombotic diagonal lesion which was ultimately treated with 2.5 mm × 12 mm Xience stent. Coronary angiogram revealed PSS in the proximal and distal LAD (red arrows).

A 64-year-old male presented with substernal chest pain an hour prior to arrival in the emergency department. Vital signs included a blood pressure of 140/81, heart rate of 69, respiration at 17 beats/min, and oxygenation at 89% on room air. Physical examination was unremarkable; however, the patient was in mild distress and diaphoretic. Electrocardiography (ECG) revealed ST elevation in leads V3-V6 (Figure 1(a)).

**Figure 2:**
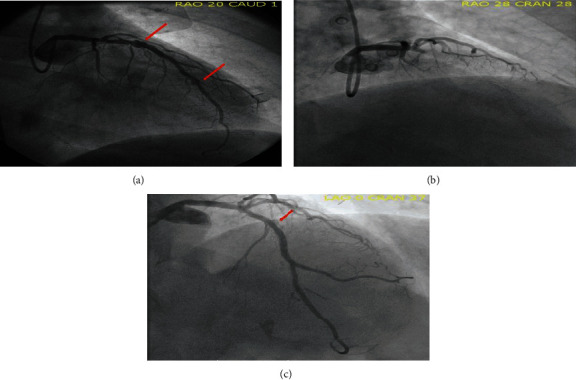
(a) 3 months later, also in 2011, the patient presented with chest pain after clopidogrel interruption prior to surgery. He was found to have acute stent thrombosis in the mid LAD and second diagonal artery. Patient was treated with angioplasty and 3.5 mm × 15 mm BMS in mid LAD. A BMS was used due to the need for an upcoming surgery. Again, PSS was seen in the proximal and distal LAD (red arrows). (b) In 2013, the patient presented with an anterior STEMI and was found to have late stent thrombosis of the proximal LAD at site of previously identified PSS. The proximal LAD lesion was treated with angioplasty, and a 2.5 mm × 23 mm BMS was placed in the distal LAD. (c) PSS was noted in the proximal LAD on final angiogram (red arrow).

Our patient has a history of coronary artery disease with multivessel percutaneous coronary intervention (PCI), heart failure with reduced ejection fraction, hypertension, and hyperlipidemia. The patient had his first myocardial infarction in 2008 in the setting of an inferior wall myocardial infarction and underwent primary PCI of the RCA. He thereafter underwent staged PCI of the circumflex and subsequent staged PCI of the left anterior descending artery (LAD) using a 3.5 mm × 28 mm Cypher stent in the proximal segment and overlapping 3 mm × 28 mm and 2.75 mm × 18 mm Cypher stents in the mid to distal LAD (Figures 1(b) and 1(c)). The patient subsequently presented in 2011 with a non-ST segment elevation myocardial infarction secondary to a thrombotic diagonal lesion which was treated using a 2.5 mm × 12 mm Xience stent. The final angiogram revealed PSS in the proximal and distal LAD (Figure 1(d)). A few months later, the patient presented with chest pain after stopping his clopidogrel prior to a procedure. He was found to have stent thrombosis within the mid LAD and second diagonal and was treated with angioplasty and a 3.5 mm × 15 mm BMS in the mid LAD. Again, PSS was noted in the proximal and distal LAD (Figure 2(a)). In 2013, the patient presented with anterior STEMI and was found to have late stent thrombosis of his proximal LAD, at the site of previous PSS (Figure 2(b)). The proximal LAD lesion was treated with balloon angioplasty, and 2.5 mm × 23 mm BMS was placed in the distal LAD. PSS was noted in the proximal LAD on final angiogram (Figure 2(c)).

**Figure 3:**
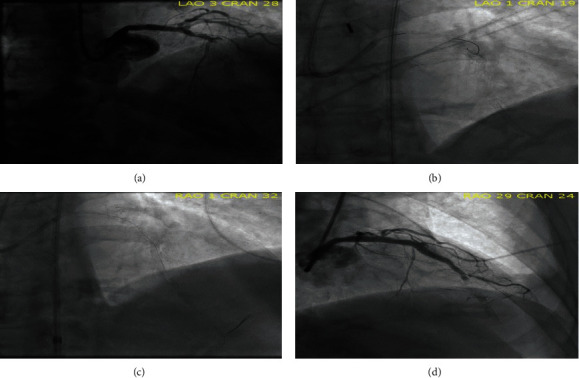
(a) During the index procedure, coronary angiogram revealed acute stent thrombosis of proximal LAD and stent fracture with aneurysm formation at site of previously identified PSS. (b) Failed attempt at advancing wire beyond fractured stent in the proximal LAD, outline the coronary aneurysm. (c) Successful crossing of Cypher stent in proximal LAD on a third attempt given recurrent symptoms. (d) Final coronary angiogram with the inability to establish TIMI 3 flow in distal LAD.

Due to the nature of the chest pain on presentation and ECG changes, the cardiac catheterization laboratory was activated. Emergent coronary angiography demonstrated acute stent thrombosis of the proximal LAD at the site of previous PSS, with evidence of stent fracture and aneurysm (Figure 3(a)). Additionally, stent fracture in the distal LAD was also demonstrated at the site of previous PSS.

The patient initially presented to an outside hospital, and despite multiple attempts, the lesion could not be wired due to the fracture and aneurysm (Figure 3(b)). An intra-aortic balloon pump (IABP) was placed, and the patient was transferred to our institution. Intravascular ultrasound (IVUS) of the proximal LAD demonstrated an undersized stent, aneurysmal dilation with intramural hematoma in the area of stent fracture (see Supplementary Material for media file—Media 1). The lesion was successfully crossed using a Sasuke microcatheter and Gaia Next wire (Ashahi, Japan); however, there was significant difficulty in passing equipment leading to wire loss and aborting the procedure. The patient was brought back a few hours later due to recurrent symptoms with successful placement of DES; however, his procedure was complicated by no reflow and overall unsuccessful revascularization (Figures 3(c) and 3(d)). After initial recovery in the hospital, the patient was safely discharged home on goal directed medical therapy. After >6 months of outpatient follow-up and uptitration of his medication, the patient had improvement of his left ventricular ejection fraction to >45% without recurrent admission to the hospital. We recommended prolonged dual antiplatelet given the patients low bleeding risk.

## 3. Discussion

PSS is defined as contrast staining outside of the stent contour extending >20% of the stent diameter measured by quantitative coronary angiography [1]. PSS has been associated with stent fracture, target lesion revascularization, and late stent thrombosis [1]. Imai et al. evaluated 3081 lesions in 1998 subjects, for angiographic evidence of PSS 12 months after sirolimus-eluting stent implantation. They found that stent fracture was more frequent in lesions with PSS (43.1% vs. 5.4%, *P* < 0.0001). Several mechanisms have been postulated to be predictors of stent fracture including stents placed in the RCA, the use of overlapping stents, longer stent length, and vessel tortuosity [2, 4]. The presence of chronic total occlusion has been identified as an independent risk factor for PSS as well [1]. On review of previous coronary angiograms of our patient, we noted PSS at the site of stent fracture and at the site of previously implanted Cypher stents. The PSS preceded the development of stent fracture. Recognizing the appearance of PSS may have led to further investigation and increase suspicion for stent failure during preceeding interventions and may have prompted use of intravascular imaging during prior interventions.

Stent fracture is a known complication of first-generation, sirolimus-eluting stents and was first reported in 2004 [4]. Several studies have reported increase in occurrence of in-stent restenosis and stent thrombosis in the setting of stent fracture [5]. It has been speculated that stent fracture results in decrease in drug availability at the location of fracture, leading to abnormal proliferation of smooth muscle lining the coronary vessel and subsequent impairment of endothelialization [6]. Furthermore, the presence of the exposed metallic structure leads to activation of inflammatory cytokines and triggers platelet activation that subsequent stent thrombosis [1, 7]. It has been hypothesized that aneurysmal dilation of coronary vessels after placement of drug eluting stents may be due to immunologic reaction from the drug which can cause intimal remodeling and dilation of the vessel [3]. Imaging techniques such as fluoroscopy with or without contrast, intravascular ultrasound, multislice CT, and optical coherence imaging are important modalities to identify stent fracture [5]. PSS can also be easily identified on by fluoroscopy and is an important marker of possible future stent failure.

## 4. Conclusion

PSS is most commonly associated with first generation sirolimus-eluting coronary stents such as the Cypher stent and can be an early marker for development of stent failure; increasing the risk of stent fracture, stent thrombosis, and in-stent restenosis.

## Data Availability

Data sharing is not applicable to this article as no datasets were generated or analysed during the current study.

## Supplementary Materials

Supplementary Materials Media 1: intravascular ultrasound (IVUS) of the proximal LAD demonstrated an undersized stent, aneurysmal dilation with intramural hematoma in the area of stent fracture. https://drive.google.com/file/d/1issR21HiqlJ2N5Uye1b1obODjG3CFklo/view?usp=sharing.

## References

[1] Imai M., Kadota K., Goto T. (2011). Incidence, risk factors, and clinical sequelae of angiographic peri-stent contrast staining after sirolimus-eluting stent implantation. *Circulation*.

[2] Nakazawa G., Finn A. V., Vorpahl M. (2009). Incidence and predictors of drug-eluting stent fracture in human coronary artery a pathologic analysis. *Journal of the American College of Cardiology*.

[3] Doi H., Maehara A., Mintz G. S. (2009). Classification and potential mechanisms of intravascular ultrasound patterns of stent fracture. *The American journal of cardiology*.

[4] Sianos G., Hofma S., Ligthart J. M. R. (2004). Stent fracture and restenosis in the drug-eluting stent era. *Catheterization and cardiovascular interventions: official journal of the Society for Cardiac Angiography & Interventions*.

[5] Chinikar M., Sadeghipour P. (2014). Coronary stent fracture: a recently appreciated phenomenon with clinical relevance. *Current cardiology reviews*.

[6] Kuramitsu S., Iwabuchi M., Haraguchi T. (2012). Incidence and clinical impact of stent fracture after everolimus-eluting stent implantation. *Circulation: Cardiovascular Interventions*.

[7] Lee M. S., Jurewitz D., Aragon J., Forrester J., Makkar R. R., Kar S. (2007). Stent fracture associated with drug-eluting stents: clinical characteristics and implications. *Catheterization and cardiovascular interventions: official journal of the Society for Cardiac Angiography & Interventions*.

