# Supplementary material for: Peri-Contrast Staining as a Marker of Stent Failure: Restenosis, Thrombosis, and Fracture
Source: Case Rep Cardiol. 2021 Sep 6;2021:4688228. doi: 10.1155/2021/4688228 (PMC8556128; doi:10.1155/2021/4688228)
Supplement: Supplementary Materials — Media 1: intravascular ultrasound (IVUS) of the proximal LAD demonstrated an undersized stent, aneurysmal dilation with intramural hematoma in the area of stent fracture. https://drive.google.com/file/d/1issR21HiqlJ2N5Uye1b1obODjG3CFklo/view?usp=sharing. [file 4688228.f1.docx]

**Supplementary Material**

Media 1: Intravascular ultrasound (IVUS) of the proximal LAD demonstrated an undersized stent, aneurysmal dilation with intramural hematoma in the area of stent fracture.

<https://drive.google.com/file/d/1TwT1h38pEKq1T_WRTxBB4uHbAddphG3D/view?usp=sharing>
